# Implementation Research to Improve Intrauterine Contraceptive Device (IUD) Uptake Among Women of Reproductive Age in Meghalaya: Insights From the Formative Phase

**DOI:** 10.7759/cureus.106130

**Published:** 2026-03-30

**Authors:** Wansalan K Shullai, Reema Mukherjee, Valerie J Laloo, Badon Shylla, Manika Agarwal, Mrunali Zode, Rutuja Patil, Sudipto Roy, Tanica Lyngdoh

**Affiliations:** 1 Obstetrics and Gynecology, North Eastern Indira Gandhi Regional Institute of Health and Medical Sciences, Shillong, IND; 2 Community Medicine, Indian Council of Medical Research, Delhi, IND; 3 Surveillance, Directorate of Health Services (Maternal and Child Health and Family Welfare), Shillong, IND; 4 Public Health, National Health Mission (NHM), Shillong, IND; 5 Reproductive, Child Health, and Nutrition (RCN), Indian Council of Medical Research, Delhi, IND; 6 Community Health Research Unit (CHRU), King Edward Memorial (KEM) Hospital Research Center, Pune, IND; 7 Vadu Rural Health Program (VRHP), King Edward Memorial (KEM) Hospital Research Center, Pune, IND; 8 Development Research, Indian Council of Medical Research, Delhi, IND; 9 Research and Development, Academy of Scientific and Innovative Research, Ghaziabad, IND

**Keywords:** attitudes, birth spacing, contraception, implementation research, intrauterine contraceptive device (iud), knowledge, matrilineal, meghalaya, practice, quantitative and mixed methods research

## Abstract

Introduction: Providing high-quality family planning services is one of the cornerstones for improving maternal and child health outcomes, and the intrauterine contraceptive device (IUD) is among the most cost-effective contraceptive methods available. Despite this, IUD use among women in Meghalaya remains very low. There is a strong need to understand the gaps in our system and monitor the delivery of services in order to optimize the existing services and ensure that the right impact is achieved in the community. To address this gap, a multiphase implementation research project, which included a Formative phase, Co-Implementation phase, and Iteration and Dissemination phases, was undertaken to increase IUD uptake. The formative phase of the study aimed to assess knowledge, attitudes, and practices (KAP) related to IUD use and to identify key barriers to its uptake.

Methods: This research study has been planned to facilitate the coming together of all stakeholders across the broad spectrum of health systems to ensure maximizing the beneficial impact of using the IUCD as a spacing device*. *The* *Formative phase utilized a mixed-methods design combining a quantitative method via a cross-sectional KAP survey (n = 1,064), along with the qualitative methods (49 in-depth interviews and three focus group discussions). A KAP survey assessed KAP; qualitative interviews and focus group discussions (FGDs; guided by the WHO Health Systems Framework) explored barriers and facilitators to IUD uptake. East Khasi Hills and Ri Bhoi districts were chosen, covering urban, semiurban, and rural settings across Subcenters, Primary Health Centers, Community Health Centers, and a tertiary hospital. A KAP survey assessed KAP; qualitative interviews and FGDs explored barriers and facilitators to IUD uptake.

Results: Awareness of contraception was high (1,015 (95.4%) and 959 (90.1%) specifically for IUDs), but only 141 (13.3%) had ever used an IUD. Common barriers included fear of side effects, myths/misconceptions, spousal/family disapproval, and inadequate counseling. IUD use was more frequent among women with more than two children and an older youngest child.

Conclusions: Despite widespread awareness, IUD uptake remains low due to persistent sociocultural barriers and health system gaps. Targeted counseling, strengthened provider training, and male engagement are essential to overcome these barriers and improve IUD uptake. Bridging this divide requires context-specific strategies that strengthen provider competence, foster community trust, and engage both men and families in open dialogue.

## Introduction

India is committed to achieving universal access to sexual and reproductive healthcare by 2030, in alignment with Sustainable Development Goal 3.7 [1]. Over the years, India’s Family Planning program has evolved significantly in both policy and implementation, aiming to stabilize the population and improve maternal and child health outcomes [2].

Poor utilization of family planning methods in Meghalaya has a direct impact on the high MMR. The current maternal mortality ratio of Meghalaya is 211/1 lakh live births as per the National Family Health Survey (NFHS)-4. The two most important and common causes of maternal mortality in Meghalaya are postpartum hemorrhage and anemia. Both of these are avoidable causes of maternal mortality related to higher order pregnancies and lack of adequate spacing between two consecutive pregnancies, thereby increasing the importance of contraceptive use. Therefore, the long-term importance of strengthening contraceptive use is to decrease the MMR. Birth spacing plays a crucial role in reducing maternal mortality by preventing unplanned pregnancies, unsafe abortions, and the health risks associated with closely spaced pregnancies [3].

It also empowers women by allowing them to space or limit childbearing, improving their educational and employment opportunities, and ensuring better health and nutrition for children [4]. Contraceptive methods like the intrauterine contraceptive device (IUD), introduced in India in 1965, have become a cost-effective solution for preventing high-risk pregnancies.

IUD use in India declined from 2.0% in the NFHS-1 (1992-1993) to 1.5% in NFHS-4 (2015-2016). With various efforts made by the health department, in NFHS-5, IUD use has increased to 2.1% (NFHS-5) [5]. Uttar Pradesh, India’s most populous state [6], continues to predominantly use traditional contraceptive methods due to preference rather than lack of availability or awareness of modern options [7]. Similarly, a Bihar study identified fear of side effects, such as headaches, menstrual disturbances, nausea from pills, and heavy bleeding after IUD or injectable use, as a key reason for nonuse of contraception [8].

In Meghalaya, historical practices shaped a matrilineal system in which ancestral property passed from mother to daughter, as men were often away at war. Women became custodians of family, culture, and property, a tradition reinforced by Khasi and Jaintia rulers entrusting households to queens, and this legacy of female empowerment continues in Khasi society today [9]. Meghalaya has a high total fertility rate of 2.9 and a substantial unmet need for contraception (26.9%); despite its matrilineal society and women’s social and economic autonomy, family planning use remains low [10].

We focused on IUD uptake in our study due to its benefits above all other available options. IUD is long-term reversible and has low failure rates. Further, the Directorate of Health Services of Meghalaya also suggested assessing the acceptability of IUD, given its system-level priority as a birth-spacing technique. In Meghalaya, around 70% of healthcare providers, including auxiliary nurse midwives (ANMs), staff nurses, and medical officers (including AYUSH), have received hands-on training in IUD insertion and counseling. Postpartum IUD training spans three days, while regular IUD insertion training lasts six days. IUDs are centrally procured through the State National Health Mission, with supply and performance monitored via the Family Planning Logistic Management Information System and Health Management Information System, and data updated by ANMs and ASHAs (Accredited Social Health Activist). Family planning messages on healthy timing and spacing of pregnancies are delivered through these frontline workers and media outreach.

Despite these efforts, IUD uptake remains low in Meghalaya, especially in East Khasi Hills, Mawryngkneng, and Ri Bhoi, which report high maternal (107/100,000) and infant (32.3/1,000) mortality (NFHS-5); scaling effective family planning interventions within health systems remains challenging [11]. Recognizing this gap, the current research is one of the initial attempts in the region aiming to design, implement, and evaluate an evidence-based implementation model to enhance IUD uptake through a phased approach.

The current study aims to increase the uptake of IUD among women in two districts of Meghalaya by a 30% increase from baseline levels. The study is being conducted in the region in four phases: 1) a formative phase to identify facilitators and barriers in existing services and utilization, 2) a co-implementation phase to develop an intervention package (Model 0) based on formative research findings and consultations with health system officials, 3) an implementation phase to implement the model in the community, and 4) an evaluation and dissemination phase to assess the intervention’s effectiveness and share findings with key stakeholders. The current paper presents findings from the formative phase of the study, which aimed to assess the knowledge, attitudes, and practices (KAP) related to contraceptive use, particularly IUD, among the target population, identify barriers to access and utilization, and explore factors that facilitate effective use through a mixed-methods approach.

## Materials and methods

Study design

A cross-sectional study was conducted using a sequential explanatory mixed-methods design, integrating quantitative and qualitative approaches. The quantitative phase was carried out first, and the findings informed the subsequent qualitative phase.

Study setting

**Figure 1:**
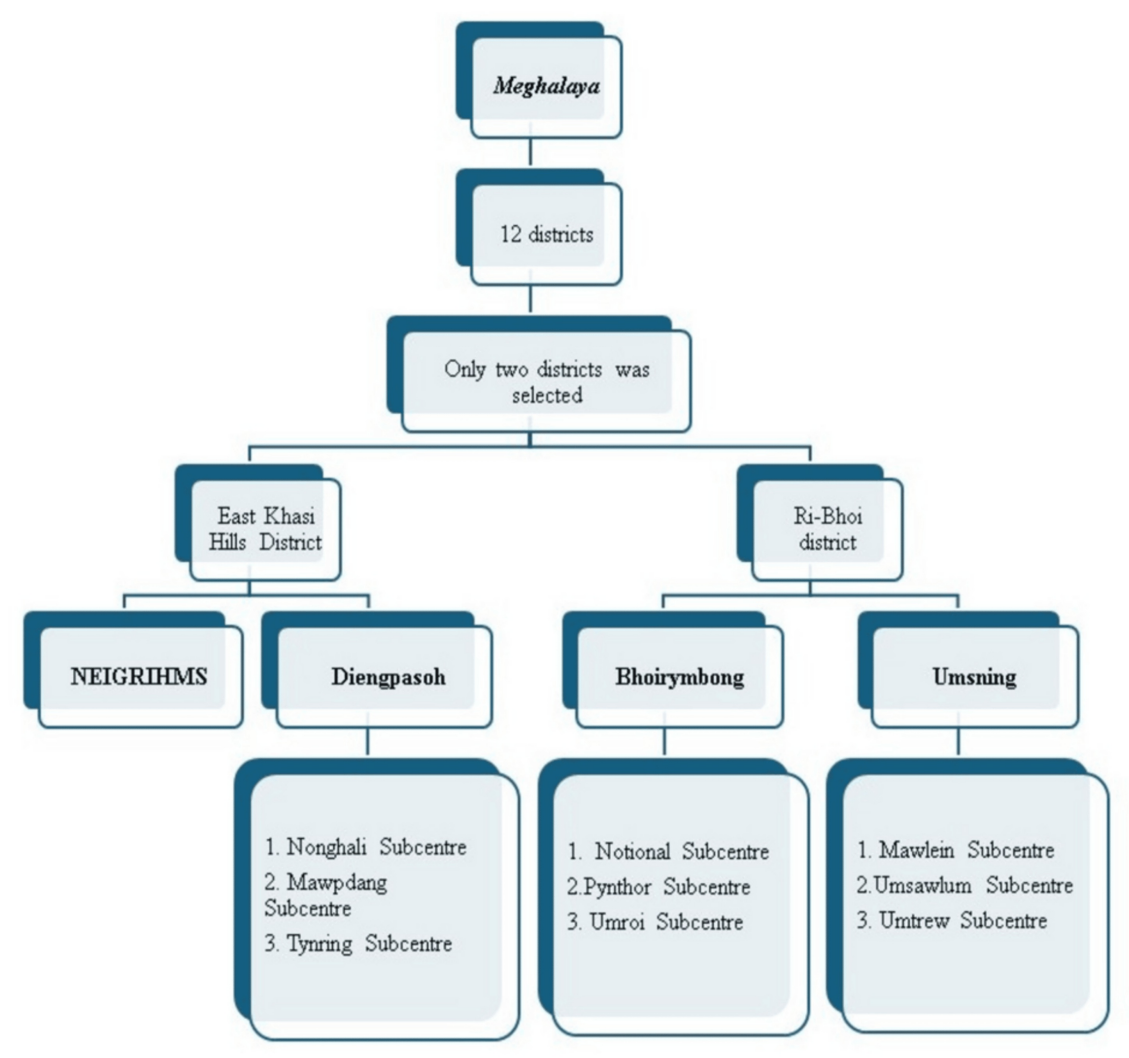
Selection of districts, blocks, and subcenters for the study

The study was implemented in two target districts, purposively chosen to include an aspirational (Ri Bhoi) and a nonpriority (East Khasi Hills) district (Figure 1). In East Khasi Hills, sites included the North-Eastern Indira Gandhi Regional Institute of Health and Medical Sciences (NEIGRIHMS) in Shillong, a tertiary referral hospital, and one rural primary health center (in Diengpasoh), along with three subcenters under its jurisdiction in the Mawryngkneng block. In Ri Bhoi district, data were collected at a subdivisional hospital (Bhoirymbong community health center (CHC) with three linked subcenters) and an additional CHC (Umsning CHC). This combination enabled the study to capture diverse population characteristics and service delivery contexts influencing IUD use across Meghalaya’s landscape.

Study duration

The study was conducted between February 26, 2024, and November 28, 2025.

Study participants, sample size, sampling method, and data management

The study participants included the following stakeholders.

Technical Support Unit

This unit includes officials from the Office of the District Medical and Health Officer (with the DMHO as the Chair). The technical support unit (TSU) helped in identifying the implementation bottleneck based on the feedback from the formative research.

Beneficiaries

An eligible couple aged 18 years or older and willing to provide informed written consent was included.

Implementers

Medical and Health Officers, Nursing Supervisors, Staff nurses, ANMs, ASHA facilitators, and ASHAs were the implementers. The implementers will implement the government program as it is and will also incorporate the identified interventions in the ongoing program.

*Research team*: The research team included the coordinating institute (NEIGRIHMS, Shillong) and the collaborating team (Directorate of Health Services, Government of Meghalaya).

*Intervention rapid learning and feedback team*: This team included the project staff. They were involved in submitting a monthly progress report on the gaps and flaws in the implementation process of the ongoing program with the TSU.

*Implementation support team*: This team included the facility public health nurse/CHO/lady health visitor (nursing supervisors) who will drive the interventions.

*Program evaluation team:* This included the project staff. They were involved in conducting the baseline and end study.

*Quantitative component: *The survey included women aged ≥18 years and written informed consent.

Sample size was calculated using NFHS-5 (2019-2020) data for Meghalaya (IUD prevalence: 4.4%) [5]. With 95% confidence and 5% precision, 920 women were needed; accounting for a 10% nonresponse rate, the target was 1,012. Ultimately, 1,064 women were enrolled. A multistage sampling approach was used: three villages per health facility catchment area were purposively selected based on distance from the subcenter, and eligible women were randomly selected from household listings or family planning registers.

Data were collected using a structured KAP questionnaire developed from national family planning guidelines (see the Appendix) [12]. Content validity was ensured through expert review. It was pilot-tested among mothers to assess clarity and feasibility, refined accordingly, and administered by trained investigators. The KAP covers sociodemographics, reproductive history, contraceptive awareness and use, perceived facilitators and barriers to IUD uptake, and knowledge of IUD benefits and side effects. The tool was translated into Khasi and Garo and back-translated. Trained female interviewers conducted face-to-face interviews at homes or health facilities. Responses were recorded on paper or tablets and entered into Microsoft Excel (Microsoft Corporation, Redmond, WA). Family planning registers and monthly reports were also reviewed to validate self-reported contraceptive use and assess service availability.

*Qualitative component: *The qualitative study included community women and healthcare providers to capture demand- and supply-side perspectives on IUD services. Focus group discussions (FGDs) involved women aged 18-49 years, with 8-10 participants per group. In-depth interviews (IDIs) were conducted with two categories of informants: 1) community women (married or recently postpartum) and 2) healthcare providers, including ASHAs, ANMs, staff nurses, and medical officers.

Participants were purposively selected to ensure variation in geographic location (urban/rural), age, parity, and contraceptive use (IUD users, other-method users, and nonusers). Data collection continued until thematic saturation, yielding 49 IDIs and three FGDs.

IDIs and FGDs were conducted using semistructured guides, informed by the WHO Health Systems Framework [13], to explore service experiences, attitudes toward IUDs, barriers to uptake, and suggestions for improving adoption. Provider guides also addressed training, counseling, supply logistics, and systemic challenges. Tools were pilot-tested and adapted iteratively. IDIs and FGDs were conducted in local languages, ensuring privacy, audio-recorded with consent, accompanied by field notes, and later transcribed and translated into English. Data collection continued until thematic saturation was reached.

*Additional assessments: *A health facility assessment was conducted to evaluate the availability of trained manpower, equipment, and space. The research team also validated the Eligible Couple registers by comparing actual data with the annual action plans prepared by the health facilities.

Data analysis

Sociodemographic characteristics and KAP variables were summarized using descriptive statistics. Continuous variables were reported as mean (SD) or median (interquartile range (IQR)), depending on the data distribution, while categorical variables were presented as frequencies and percentages. Univariable logistic regression analyses were conducted to examine factors associated with current IUD use and future preference to use an IUD. Variables showing statistical significance (p value less than 0.05) in univariable analyses were subsequently included in multivariable logistic regression models to identify independent predictors of IUD use and intention to use an IUD in the future, respectively. Results were reported as adjusted odds ratios (AORs) with 95% CIs. All statistical analyses were performed using STATA, version 15.1 (StataCorp LLC, College Station, TX). Qualitative data from IDIs and FGDs underwent rapid thematic analysis, including transcript review, independent coding, and synthesis using matrix charting aligned with WHO health-system domains. Finally, quantitative, qualitative, and facility-assessment findings were triangulated to examine convergence and complementarity across data sources, providing a comprehensive understanding of factors influencing IUD uptake.

## Results

A total of 1,064 mothers participated in the study. Over half (51.1%) were aged 26-35 years, and 26.3% were 18-25 years (Table 1). Nearly half belonged to the Bhoi community (49.2%), followed by Khasi (40.0%), and the majority identified as Christian (91.4%). Most participants were homemakers (66.7%), with 30.1% employed and 3.2% students, retired, or unemployed. The highest level of education varied, with 23.3% having completed secondary education and 13.1% graduation or higher, and only 8.0% had no formal schooling. Over half of households reported a monthly income of ₹10,001-15,000 (58.2%), and most families were nuclear (68.5%).

**Table 1:** Sociodemographic characteristics of the participants (n = 1,064) INR: Indian rupee; IQR: interquartile range

Category, n (%)	Value (n = 1,064)
Age of mother	
18-25 years	280 (26.3)
26-35 years	544 (51.1)
36-49 years	240 (22.6)
Ethnicity	
Bhoi	523 (49.2)
Khasi	426 (40.0)
Pnar	19 (1.8)
War	8 (0.8)
Others	88 (8.3)
Religion	
Christian	973 (91.4)
Hindu	61 (5.7)
Niam Tynrai	26 (2.4)
Muslim	4 (0.4)
Occupation	
Homemaker	710 (66.7)
Employed	320 (30.1)
Unemployed/retired/student	34 (3.20)
Education	
No formal schooling	85 (8.0)
Lower primary	210 (19.7)
Upper primary	239 (22.5)
Secondary	248 (23.3)
Higher secondary	143 (13.4)
Graduation and above	139 (13.06)
Monthly income of the head of the family (INR)	
<10,000	240 (22.6)
10,001-15,000	619 (58.2)
More than 15,000	205 (19.3)
Type of family	
Nuclear	729 (68.5)
Joint	335 (31.5)
Number of children	
0	79 (7.4)
1	279 (26.2)
2	245 (23.0)
3	194 (18.2)
4	120 (11.3)
≥5	147 (13.8)
Median number of children (IQR)	2 (1, 4)
Number of deliveries (n = 1,064)	
0	74 (7.0)
1	237 (22.2)
2	231 (21.7)
3	190 (17.9)
4	123 (11.6)
≥5	209 (19.6)
Age gap with last child (n = 1,064)	
Less than 1 year	356 (33.5)
1-2 years	263 (24.7)
2-3 years	116 (10.9)
More than 3 years	329 (30.9)

Regarding reproductive characteristics, the median number of children was two (IQR: 1-4). About one-fourth (26.2%) of mothers had a single child, while 13.8% had five or more. Similarly, 22.2% had one delivery and 19.6% had five or more. The interpregnancy interval was less than one year for 33.5% of participants, one to two years for 24.7%, and more than three years for 30.9%.

Knowledge and awareness of contraception (in particular, IUDs)

Table 2 shows KAP related to IUD use among 1,064 participants. Most respondents were aware of birth spacing (95.2%), contraception (95.4%), and the IUD (90.1%). Healthcare workers reported similarly high awareness, noting that ASHAs routinely counsel new mothers on the benefits of birth spacing for maternal and child health.

**Table 2:** Knowledge, attitude, and practice related to IUD among participants (n = 1,064) ^*^The “Other” category of response for “Reason for choice of contraceptive” consisted mainly of the reason “as advised by a healthcare professional” ^#^The question was a multiple-response type IUD: intrauterine contraceptive device; ASHA: Accredited Social Health Activist

Variable	Category	n (%)
Knowledge		
Knowledge about birth spacing	Have knowledge	1,013 (95.2)
	Does not have knowledge	51 (4.8)
Awareness session/campaign on birth spacing	Held	875 (82.2)
	Not held	189 (17.8)
Knowledge about contraception	Have knowledge	1,015 (95.4)
	Does not have knowledge	49 (4.6)
Knowledge about IUD	Have knowledge	959 (90.1)
	Does not have knowledge	105 (9.9)
Source of knowledge about IUD (n = 959)	Healthcare worker	484 (50.5)
	ASHA	276 (28.8)
	Friends/relatives	134 (14.0)
	Newspaper/media	19 (2.0)
	Others	46 (4.8)
Attitude		
Received counseling regarding IUD during pregnancy/postpartum period	Received	701 (65.9)
	Not received	363 (34.1)
Preference for IUD in the future	Will not prefer	606 (57.0)
	Not sure	308 (29.0)
	Will prefer	150 (14.1)
Reason for not using IUD^#^ (n = 923)	Fear of side effects	348 (37.7)
	No information about IUD	173 (18.7)
	Don not want to use IUD	188 (20.4)
	Belief that the child is God’s gift	36 (3.9)
	History of medical complications	28 (3.0)
	Husband refusal	19 (2.0)
	Relative refusal	11 (1.2)
	Far distance of health facility	2 (0.2)
	Others	123 (13.3)
Practice		
Used any contraception	Used	564 (53.0)
	Have not used	500 (47.0)
Type of contraceptive used (n = 564)	O.C. pills	209 (37.1)
	IUD	141 (25.0)
	Injectable contraceptive	130 (23.1)
	Rhythm/natural method	128 (22.7)
	Condoms	65 (11.5)
	Others	7 (1.2)
Reason for choice of contraceptive (n = 564)	Easy to use	235 (41.7)
	Safer than others	142 (25.2)
	Easily available	74 (13.1)
	Long lasting	32 (5.7)
	No discomfort	31 (5.5)
	No pain	27 (4.8)
	Others	58 (10.3)*
Used IUD	Used	141 (13.3)
	Not used	923 (86.7)
Time of IUD insertion (n = 141)	Post-partum	92 (65.3)
	Other time	47 (33.3)
	Postabortion	2 (1.4)
Duration of IUD in-utero (n = 141)	More than one year	74 (52.5)
	One year or less	51 (36.2)
	One year	16 (11.3)
Had any complaints using IUD (n = 141)	Yes	70 (49.6)
	No	71 (50.4)
Type of complaints experienced (n = 70)	Cramping and pain	15 (21.4)
	Heavy bleeding	11 (15.7)
	Improper position	6 (8.6)
	Partial/complete expulsion	6 (8.6)
	Menstrual irregularities	4 (5.7)
	Infection	2 (2.9)
	Uterine perforation	1 (1.4)
	Pregnancy with IUD in utero	1 (1.4)
	Others	24 (34.3)
Continue to use IUD (n = 141)	Yes	40 (28.4)
	No	101 (71.6)
Reason for removal of IUD (n = 101)	Discomfort	9 (8.9)
	Thread came out	7 (6.9)
	Not needed anymore	8 (7.9)
	Infection	3 (3.0)
	Loss of thread	3 (3.0)
	Influenced by others’ negative comments	2 (2.0)
	Others	69 (68.3)

One ASHA worker explained, “When I visit new mothers, especially those with their first child, I explain the importance of family planning and spacing births. Having children every year can affect the mother’s health, the baby’s growth, and even the family’s finances, food, and schooling. Those who understand come back for more guidance, and so far, eight women are using oral contraceptive pills (OCPs), and one is using Antara.”

Women themselves acknowledged that spacing allows them to regain health and care for their children: “What I understand is that it is for the benefit of us mothers when it comes to our health and well-being. And also, for the children, when we give birth too quickly, we don’t get to give love and attention to them.” (Mother)

About 82.2% reported that awareness sessions or campaigns had been conducted in their area. This was confirmed in the qualitative interviews that health workers “used to come once a month for immunization and also give awareness about birth spacing” (Community Member). However, not all women were reached: one mother from a remote village admitted, “I don’t get proper awareness about birth spacing… maybe they came and I did not attend”, indicating gaps in communication despite community outreach."

While general awareness was high, detailed knowledge about IUDs was limited. Health workers reported that many women had heard of IUDs but did not fully understand them. An ASHA noted that “I was trained to counsel that IUD does not affect the health, and it is safer than others. After insertion, there will be pain, but it will not last long.”

The most frequently cited sources of IUD-related information were healthcare workers (50.5%) and ASHAs (28.8%), followed by friends or relatives (14.0%) and media (2.0%). In interviews, several women confirmed hearing about IUDs from nurses or ASHAs. Despite this, many women were fearful about using it. Myths persisted that strenuous physical activity could dislodge or move an IUD inside the body. For example, one mother expressed: “I don’t know much about IUD, I am scared to use it since I have to do heavy work in the fields, lifting heavy objects.” Another woman shared her anxiety about the device’s presence: “We are scared because if we go to the river to wash clothes, we carry heavy load and if by mistake anything happens because the thing is inside…it’s frightening.”

Attitudes and perceptions regarding IUD use

Despite high awareness, attitudes toward IUD use remained cautious (Table 2). Although 66% received counseling, most participants were unwilling (57.0%) or unsure (29.0%) about future use, with only 14.1% willing. Among nonusers, fear of side effects (37.7%), unwillingness (20.4%), and lack of information (18.7%) were the main reasons. This was also mirrored in the qualitative findings. A medical officer remarked that “Copper-T… most of them are like it’s a horrible thing.” Another medical officer said, “They do not prefer IUD; they prefer other methods, mostly injectables and OCP.”

Women held a preconceived notion that an IUD is harmful, sometimes attributing unrelated symptoms to the device. “Some women experienced complications earlier and also abdominal pain after insertion and they tend to attribute it to the IUD, even when it might not be the cause. The mindset here is that any problem is blamed on the IUD. Another reason could be that people don’t fully understand it yet, so we really need more awareness and counseling programmes to explain its benefits.” (Medical Officer)

Further, 3.9% of survey respondents attributed nonuse to the belief that “the child is God’s gift.” Health workers noted that some families view contraception as interfering with God’s will: “They say that it is the will of God to have children” (Staff nurse). A few women echoed this sentiment; one mother hesitated to use contraception because others had scared her that each child was destined to come into the world.

Partner and family influences emerged as critical factors shaping attitudes toward IUD use. While only 3.2% women in the survey explicitly blamed husband or family opposition for avoiding IUDs, interviews suggest that spousal consent and support are critical. As one participant explained: “I need his agreement… I cannot take the decision without my husband’s consent.” Providers observed that some women “require consent of their family members” and face pressure from in-laws not to use a CuT (IUD). Further, a staff nurse noted that lack of spousal support often influenced contraceptive choices: “Many women choose injections or OCPs instead of CuT because their husbands don’t support them. They prefer methods that can be used without their husband’s knowledge.” Several ASHAs confirmed this pattern: “Most of the women here prefer OCP or Antara (injectable)…they felt scared using IUD.” (ASHA).

Contraceptive practices and IUD uptake

Regarding contraceptive practice (Table 2), 53.0% had ever used a method, with oral pills most common (37.1%), followed by IUDs (25.0%), injectables (23.1%), and rhythm/natural methods (22.7%). The qualitative findings corroborated this pattern of preference. Health personnel consistently reported that women tend to opt for injectables or pills over IUDs. According to one medical officer, “The most used contraceptive is Antara (injectable). It is easier and simpler; it can be given at the village level, whereas IUD requires the facility.” Further, the fact that injectables and pills were more convenient to use, require no procedure, and can be obtained more privately was a theme that emerged repeatedly in the interviews. “They felt that OCP is easy to use and causes no side effects, and thus most preferred OCP and injection” (ASHA). In contrast, IUDs were often seen as uncomfortable or inconvenient, due to the need for an insertion and the presence of the thread in the body. Notably, at the tertiary hospital site where immediate postpartum insertions were available, a staff nurse observed higher acceptance: “Here in this hospital, most patients prefer CuT.”

Contraceptive choice was mainly influenced by ease of use (41.7%), perceived safety (25.2%), and availability (13.1%). Only 13.3% had ever used an IUD; among these, 65.3% had postpartum insertion and 52.5% retained it for over one year.

Importantly, continuation rates were low; only 28.4% of the women who ever used an IUD were still using one. Qualitative interviews provided insight into why sustaining IUD use was challenging. Approximately half (49.6%) experienced complaints such as cramping or pain (21.4%), heavy bleeding (15.7%), or expulsion (8.6%). Even minor symptoms sometimes led women to attribute health issues to the IUD. Medical officers noted that “Some patients have inserted, but they are not happy. They always come with that mindset… complaining of backache, headache, lower abdomen pain, and minor complaints” (Medical Officer).

Among IUD removals, 9% were due to personal discomfort, 7% to the string coming out, and fewer to reasons such as no longer needing contraception or fear of infection; most removals (68.3%) occurred for other reasons. Qualitative interviews provided insights into these “other” cases as being guided by healthcare advice or community narratives. This may reflect providers recommending removal due to side effects, medical reasons, or upon the patient’s request. In one instance, as shared by an ASHA, “In one case, a woman who had a CuT inserted at a district hospital became pregnant, and it was later found that the device had gone missing. News of this spread through the village, making others fearful. Since then, most prefer OCPs or Antara.” This was also in line with findings from the survey where 2% of discontinuers said they were “influenced by others’ negative comments,” highlighting how one person’s bad experience can spread through the community reinforcing fears. On the other hand, one woman mentioned that “my mother suggested me to use IUD since it is easy to use and safe compared to injectables and OCP”, indicating that family members and successful users can act as facilitators.

The qualitative interviews revealed that supply and service factors also played a role in contraceptive use patterns. Most health providers reported that while IUD commodities were readily available at their facilities with no stock-outs, the supplies of other methods, particularly the Antara injectable, had been inconsistent. Clinic staff confirmed periodic shortages of injectables, leading to a situation where “we have to ask patients to buy contraceptives themselves” or women discontinuing when stocks ran out. Further, there were also gaps in provider training and communication that could impact IUD uptake. Some nurses and mid-level providers had not received recent training in IUD insertion, meaning only doctors performed insertions in certain facilities. Providers mentioned the need to use simple language and culturally appropriate counseling to dispel myths. For example, being able to counsel in the local language (Khasi) was seen as crucial to overcoming the language barrier and ensuring women understand the benefits of IUDs.

Factors associated with IUD uptake

IUD uptake was found to be associated with maternal age (borderline significance), parity, and age of the last child (Table 3). Women aged 26-35 years (AOR = 1.68; 95% CI: 1.05-2.67) and 18-25 years (AOR = 1.23; 95% CI: 0.61-1.68) were more likely to use an IUD compared with those aged ≥36 years. Parity showed a strong independent association: women with more than two children were almost twice as likely to use an IUD as those with two or fewer (AOR = 1.86; 95% CI: 1.23-2.80; p = 0.003). Similarly, the age of the youngest child showed a clear positive gradient: as the child’s age increased, the odds of IUD use also increased. The qualitative data supported this pattern: some women indicated they delayed IUD acceptance until they had achieved their desired family size or had children of both genders. One mother admitted she initially avoided using an IUD due to fear, but after having several children and facing difficulties, she “decided to use contraceptives since it has been really difficult for me.” Notably, although religion was not associated with IUD uptake, a few participants in interviews mentioned religious or cultural objections as personal deterrents.

**Table 3:** Factors associated with IUD use (n = 1,064) ^*^p values in the multivariable model represent global p values obtained from Wald tests assessing the overall association of each categorical variable with the outcome in the multivariable model. Category-specific p values were not calculated in the multivariable model by design IUD: intrauterine contraceptive device; OR: odds ratio; CI: confidence interval

Categories	Total (n = 1,064)	n (%) used IUD	Univariable analysis, crude OR (95% CI)	p value	Multivariable model, adjusted OR (95% CI)	p value^*^
Age of the mother						
18-25 years	280	19 (6.79)	0.38 (0.21, 0.67)	0.001	1.23 (0.61, 2.49)	-
26-35 years	544	83 (15.26)	0.93 (0.61, 1.41)	0.72	1.68 (1.05, 2.67)	-
36 years and more	240	39 (16.25)	Reference	-	Reference	0.064
Religion						
Christian	973	129 (13.26)	Reference	-	-	-
Hindu	61	7 (11.48)	0.85 (0.38, 1.9)	0.69	-	-
Muslim/Niam	30	5 (16.67)	1.31 (0.49, 3.48)	0.59	-	-
Ethnicity						
Khasi	426	52 (12.21)	Reference	-	-	-
Bhoi	523	76 (14.53)	1.22 (0.84, 1.79)	0.30	-	-
Other	115	13 (11.3)	0.92 (0.48, 1.75)	0.79	-	-
Education						
No formal schooling	85	7 (8.24)	0.69 (0.27, 1.75)	0.43	-	-
Lower to upper primary	449	58 (12.9)	1.14 (0.63, 2.06)	0.66	-	-
Secondary schooling	248	38 (15.32)	1.39 (0.75, 2.6)	0.30	-	-
Higher secondary schooling	143	22 (15.38)	1.4 (0.7, 2.79)	0.34	-	-
Graduate and above	139	16 (11.51)	Reference	-	-	-
Occupation						
Employed	320	46 (14.37)	1.15 (0.79, 1.68)	0.48	-	-
Homemaker/unemployed	744	95 (12.77)	Reference	-	-	-
Monthly income of the head of the family						
<10,000	240	33 (13.75)	0.75(0.45, 1.25)	0.27	0.74 (0.43, 1.28)	-
10,001-15,000	619	72 (11.63)	0.62(0.40, 0.96)	0.03	0.58 (0.36, 0.93)	-
>15,000	205	36 (17.56)	Reference	-	Reference	0.072
Type of family						
Joint	335	33 (9.85)	Reference	-	Reference	-
Nuclear	729	108 (14.81)	1.59 (1.05, 2.40)	0.03	1.23 (0.78, 1.92)	0.372
No of children						
≤2	603	62 (10.28)	Reference	-	Reference	-
>2	461	79 (17.14)	1.80 (1.26, 2.58)	0.001	1.86 (1.23, 2.8)	0.003
Age of the last child						
Less than a year	356	28 (7.87)	Reference	-	Reference	<0.001
1-2	263	27(10.27)	1.34 (0.77, 2.33)	0.30	1.24 (0.7, 2.18)	-
>2 to 5	246	32 (13.01)	1.75(1.03, 2.99)	0.04	1.6 (0.92, 2.78)	-
>5	199	54 (27.14)	4.36 (2.66, 7.17)	<0.001	4.33 (2.48, 7.56)	-

Factors influencing future preference for IUD uptake

Table 4 shows that maternal age and ethnicity were independently associated with future preference for IUD use. After adjustment, women aged 18-25 years (AOR = 2.15; 95% CI: 1.16-3.99; p = 0.04) and 26-35 years (AOR = 1.96; 95% CI: 1.12-3.41) had significantly higher odds of preferring IUD use in the future compared with women aged ≥36 years. Qualitative findings indicated that many younger women were interested in effective spacing methods but delayed adoption due to fear, misinformation, or lack of confidence. Women from the Bhoi community were more likely to express future IUD preference than Khasi women (AOR = 1.67; 95% CI: 1.13-2.46). Providers reported greater receptivity to counseling among Bhoi women, while some Khasi women expressed concerns related to side effects and heavy physical work. Although the age of the youngest child was significant in univariable analysis, this association did not persist after adjustment. No significant associations were observed for religion, education, occupation, income, family type, or parity. Overall, these findings indicate that future preference for IUD use is influenced more by perceived readiness, counseling experiences, and community narratives than by socioeconomic characteristics alone.

**Table 4:** Factors influencing future preference of IUD ^*^p values in the multivariable model represent global p values obtained from Wald tests assessing the overall association of each categorical variable with the outcome in the multivariable model. Category-specific p values were not calculated in the multivariable model by design IUD: intrauterine contraceptive device; OR: odds ratio; CI: confidence interval

Categories	Total (n = 1,064)	n (%) future preference for IUD	Univariable analysis, crude OR (95% CI)	p value	Multivariable model, adjusted OR (95% CI)	p value^*^
Age of the mother						
18-25 years	280	49 (17.50)	2.62 (1.48, 4.63)	0.001	2.15 (1.16, 3.99)	-
26-35 years	544	83 (15.26)	2.22 (1.30, 3.79)	0.003	1.96 (1.12, 3.41)	-
36 years and more	240	18 (7.50)	Reference	-	Reference	0.04
Religion						
Christian	973	139 (14.29)	1.21 (0.63, 2.33)	0.57	-	-
Hindu/Muslim/Niam	61	11 (12.09)	Reference	-	-	-
Ethnicity						
Khasi	426	46 (10.80)	Reference	-	Reference	0.03
Bhoi	523	86 (16.44)	1.62 (1.11, 2.39)	0.01	1.67 (1.13, 2.46)	-
Other	115	18 (15.65)	1.53 (0.85, 2.76)	0.15	1.61 (0.88, 2.93)	-
Education						
No formal schooling	85	5 (5.88)	0.42 (0.15, 1.18)	0.10	-	-
Lower to upper primary	449	63 (14.03)	1.10 (0.63, 1.93)	0.75	-	-
Secondary schooling	248	45 (18.15)	1.49 (0.83, 2.69)	0.19	-	-
Higher secondary schooling	143	19 (13.29)	1.03 (0.52, 2.06)	0.93	-	-
Graduate and above	139	18 (12.95)	Reference	-	-	-
Occupation						
Employed	320	42 (13.13)	0.89 (0.61, 1.30)	0.55	-	-
Homemaker/unemployed	744	108 (14.52)	Reference	-	-	-
Monthly income of the head of the family						
<10,000	240	42 (17.50)	1.29 (0.77, 2.15)	0.34	-	-
10,001-15,000	619	79 (12.76)	0.89 (0.56, 1.40)	0.61	-	-
>15,000	205	29 (14.15)	Reference	-	-	-
Type of family						
Joint	335	53 (15.82)	Reference	-	-	-
Nuclear	729	97 (13.31)	0.82 (0.57, 1.17)	0.27	-	-
No of children						
≤2	603	91 (15.09)	Reference	-	-	-
>2	461	59 (12.80)	0.83 (0.58, 1.18)	0.29	-	-
Age of the last child						
Less than a year	356	63 (17.70)	2.30 (1.31, 4.06)	0.004	1.77 (0.96, 3.26)	-
1-2	263	39 (14.83)	1.86 (1.02, 3.40)	0.04	1.46 (0.77, 2.76)	-
>2 to 5	246	31 (12.60)	1.54 (0.83, 2.88)	0.17	1.32 (0.69, 2.51)	-
>5	199	17 (8.54)	Reference	-	Reference	0.28

## Discussion

This mixed-methods study in Meghalaya found near-universal awareness of contraception (95.4%) and IUDs (90.1%), but modest modern method use (53.0%) and low IUD uptake (13.3%). Pills and injectables were preferred due to ease, availability, and privacy, while fears of pain, side effects, and device movement limited IUD acceptance. IUD use was higher among older, multiparous women, with no association with education or income. This was consistent with the qualitative findings highlighting the role of persistent myths, spousal/family influence, and gaps in counseling. These findings highlight a clear gap between awareness and actual use of contraception, shaped by behavioral, cultural, and health-system factors that hinder the translation of knowledge into consistent practice.

This study points out a mismatch that is commonly observed in many low- and middle-income settings where awareness of contraception is high, yet its actual use remains limited. A similar pattern was also reported by NFHS-5, where, although knowledge of contraceptive methods is nearly universal in Meghalaya, only 27% of women reported using any method. Comparable findings have also been reported in other smaller studies from this region [14,15] and in other states such as New Delhi [16], Assam [17], and Haryana [18]. These findings suggest that awareness alone does not lead to behavior change unless the deeper social, cultural, and system-related barriers are also addressed.

The determinants of IUD use in Meghalaya appear to be multifactorial. It was observed that women with higher parity and older age were more likely to use an IUD, suggesting that long-acting reversible methods are adopted once family size is considered complete. Qualitative data supported this trend; many women preferred short-term methods until they had enough children, reflecting the influence of family norms and perceived reproductive expectations. Younger women tended to avoid contraception, probably because of family pressure to reproduce and proliferate their family. These findings highlight the need to reposition the IUD not merely as a spacing method but as an option suitable across the reproductive life course.

Fear of side effects, myths, and misinformation were major deterrents in this study. Comparative studies done in other states like Maharashtra [19] and Odisha [20] also found fear of side effects to be a major deterrent to IUD acceptance. Of those who had an IUD inserted, the majority of them gave medical problems as a reason for removal, such as conceiving even with an IUD inserted. In Bhopal, the main reason for removal is menstruation before the due date [8]. Both users and providers mentioned concerns about pain, bleeding, and the perceived risk of displacement or infection. Community narratives driven by isolated cases of failed insertions or pregnancy with an IUD in situ spread quickly and reinforce mistrust. On the other hand, facilities where providers were proactive in counseling reported higher acceptance, emphasizing the importance of provider competence and communication. Although nearly 70% of providers had received IUD training, qualitative data revealed limited refresher sessions and inadequate supervision. Strengthening provider confidence through continuous training and supportive supervision could therefore improve both the quality of counseling and user trust. A comparative study also suggests that with intense and thorough training in IUD counseling as well as insertions, IUD acceptance will be greatly enhanced, along with a decrease in medical complications associated with it [21].

Interestingly, despite Meghalaya's matrilineal structure, women’s choices were often shaped by their husbands and extended families. Many described the need for their partner’s consent or the desire to use discreet methods such as injectables or oral pills. A similar study was found where the majority of the participants did not opt for IUD usage due to husbands’ and family members' opposition [20]. Engaging men in family planning communication remains essential even in societies perceived as women-centric. Evidence from other contexts shows that involving men can improve couple dialogue and increase uptake of long-acting methods without undermining women’s autonomy. Family endorsement also played a facilitative role, as women who saw trusted relatives using IUDs were more likely to adopt them. These findings echo global literature underscoring the effectiveness of community-based and peer-led interventions in improving contraceptive acceptance.

Service accessibility further shaped contraceptive behavior. Women living closer to health centers (within 3 km) were more likely to use contraception, suggesting that even moderate distance can discourage utilization, particularly for methods requiring a procedure such as IUD insertion. Making services available closer to communities, particularly in lower level health facilities, may encourage clients to seek care for side effects and, in turn, support continued method use. This highlights the need to extend IUD services to lower levels of the health system, positioning them closer to clients to enhance access to high-quality contraceptive care and support sustained method use [22]. Interestingly, education did not emerge as a strong determinant of contraceptive use, indicating that awareness has already penetrated across educational levels. This finding suggests that the current challenge lies less in disseminating information and more in addressing psychological, social, and service-level barriers. Similarly, women in nuclear families were more likely to use contraception than those in joint families, possibly reflecting greater decision-making autonomy.

Limitations

This study collected data at a single point in time, so it can identify associations but cannot establish cause and effect. Information on contraceptive use was self-reported, which may be affected by recall errors or reluctance to share personal details. The study was conducted in only two districts of Meghalaya, limiting generalizability to other regions. The qualitative findings help explain results but are not representative of all women. Finally, the study mainly captured women’s perspectives, without including the views of husbands or other family members.

## Conclusions

Despite high awareness and the availability of services, IUD use in Meghalaya remains low due to persistent sociocultural beliefs, misinformation, and gaps in counseling and system support. Bridging this divide requires more than awareness; it calls for context-specific strategies that strengthen provider competence, foster community trust, and engage both men and families in open dialogue.

Policy efforts should move beyond awareness campaigns to models that build confidence and continuity, such as integrating IUD counseling into routine postpartum and immunization visits, using local champions to normalize discussions, and ensuring consistent follow-up to reduce discontinuation.

At the same time, collective efforts from community leaders and religious institutions will go a long way to dispel myths and reinforce positive narratives around contraception. Ultimately, improving IUD uptake in Meghalaya is less about access and more about acceptance and about reframing contraception as a means to health, empowerment and family well-being while promoting inclusive and informed family planning that engages both married and unmarried individuals.

## Appendices

I. Quantitative questionnaire

Section I: Demographic Profile of Participant

 UNIQUE ID: IUD-                                                                                                                                         Date:

 Site:

 1.     Name:

2.     Age:                                       

3.     Father/Husband’s name:

4.     Marital status:

       a)    Never married

       b)    Married (Including Co-inhabiting)

       c)    Widowed

5.     Address:

6.     Phone No.:

7.     Ethnicity:

      a)     Khasi

      b)     Pnar

      c)     Bhoi

      d)     War      

      e)     Others                                                

8.     Religion:

      a)     Christian

      b)     Hindu

      c)     Muslim

      d)     Niam Tynrai

      e)     Others

9.     Occupation:

      a)     Homemaker

      b)     Employed

      c)     Unemployed/Retired/Student

10.  Educational Level:

     a)     No formal schooling

     b)     Lower Primary

     c)     Upper Primary

     d)     Secondary school

     e)     Higher secondary school

     f)      Graduate and above

11.  Monthly income of the head of the family:

     a)     <10,000 INR

     b)     10,001-15,000 INR

     c)     Above 15,000 INR

12.  Types of family:

     a)     Joint

     b)     Nuclear

13.  No. of living children:

      a)     0

      b)     1

      c)     2

      d)     3

      e)     4

       f)      ≥5

14.  No of deliveries:

      a)     0

      b)     1

      c)     2

      d)     3

      e)     4

      f)      ≥5

15.  Age of last child:

     a)     <1 year

     b)     1-2 years

     c)     2-3 years

     d)     >3 years

Section II: Knowledge, Attitude, and Practices

1.     Do you know about birth spacing? (probe)

                         i.          Yes

                        ii.          No

2.     Is there any awareness of birth spacing in your community?

                          i.          Yes

                         ii.          No

3.     If yes, how many times was it held?

                          i.          ___________________ weeks/months

                         ii.          Cannot recall

                         iii.          Don’t know

4.     Are you planning to have more children?

                           i.             Yes

                           ii.             No

                          iii.             Maybe

5.     How much time gap are you planning to have between children?                                        

                            i.          1 yr

                           ii.          2 yrs

                          iii.          3 yrs

                          iv.          4 yrs

                           v.          >5 yrs

6.     Was your previous pregnancy planned?

                            i.     Yes

                            ii.     No      

7.     Where did you deliver your previous child?

                           i.        Ganesh Das

                           ii.        NEIGRIHMS

                          iii.        Civil hospitals

                          iv.        Private hospitals

                          v.        PHC

                         vi.        CHC

                        vii.        Home

8.   What is the reason for choosing the above facility?

                           i.       Good medical practitioners

                          ii.       Nearest

                          iii.       Well equipped

                          iv.       Familiar

                          v.       Affordable

                         vi.       Referral

                         vii.       Others (Specify)                               

9.     Do you know what contraception is? (probe) (If No, skip to Q. 13, 14 & 15)

                           i.          Yes

                          ii.          No

10.  Have you used any contraceptive method?

                           i.          Yes

                          ii.          No

11.  Which contraceptive method have you used?

                          i.             Natural method (calendar method/pulling out method)

                         ii.             Condom

                        iii.             Injectables

                        iv.             IUD

                         v.             Oral Contraceptive Pills (OCP)

                        vi.             Others (specify)

12.  Why did you choose this particular contraceptive method?

                          i.             Easily available

                          ii.             Easy to use

                         iii.             No discomfort

                         iv.             No pain

                          v.             Safer than others

                         vi.             Long lasting

                        vii.             Others (Please specify)

13.  Do ASHAs/healthcare workers visit your home and provide information about contraception/birth spacing?

                          i.          Yes

                          ii.          No

                         iii.          Don’t know

14.  If yes, how many times do they visit?

                          i.          ___________________ weeks/months

                         ii.          Cannot recall

                        iii.          Don’t know

15.  Do you know about IUDs? (If no, skip to 17 and 19)

                          i.          Yes

                         ii.          No

16.  If yes, from where?   :

                          i.          Friends/Relatives

                          ii.          ASHA

                         iii.          Healthcare workers

                         iv.          Media/Newspapers

                          v.          Others (Please specify)                

17.  During or after pregnancy/delivery, did you receive any counselling from health workers about the IUD?

                           i.          Yes

                          ii.          No

18.  Have you ever used an IUD?

                           i.          Yes

                          ii.          No

19.  If not using IUD, please mention why (Skip to 30 and 31)

                           i.          Fear of side effects

                          ii.          History of medical complications

                         iii.          Husband's refusal

                         iv.          Relative refusal

                          v.          God’s gift

                          vi.          Far distance of the health facility

                         vii.          Did not get information

                        viii.          Others (please specify)

20.  If yes, what type?

                           i.         Cu T 375 (5 years)

                           ii.         Cu T 380A (10 years)

21.  When was the IUD inserted?

                          i.          Postabortion

                          ii.          Postpartum

                         iii.          Other time

22.  Are you still using the IUD?

                         i.          Yes

                        ii.          No

23.  If No, reason for stopping?

                         i.          Thread came out

                         ii.          Loss of thread

                        iii.          Infection

                        iv.          Discomfort

                         v.          Not needed anymore

                        vi.          Influenced by others’ negative comments

                       vii.           Others (please specify)

24.  For how long have you been using the IUD?

                         i.          Less than a year

                         ii.          One year

                        iii.          More than one year

25.  Have you ever had any problems using an IUD?

                          i.          Yes

                         ii.          No

26.   If yes, what are the problems experienced?

                         i.          Heavy bleeding

                        ii.          Menstrual irregularities

                       iii.          Cramping and pain

                       iv.          Infection

                        v.          Improper position

                       vi.          Partial or complete expulsion of IUD

                      vii.          Pregnancy with an IUD in place

                     viii.          Uterine perforation

                      ix.          Others (specify)                                                                                                                                             

27.   From where do you procure an IUD?          :

                        i.          NEIGRIHMS

                       ii.          Ganesh Das Hospital

                      iii.          Private hospitals

                      iv.          Civil hospitals

                       v.          PHC

                      vi.          CHC

                     vii.          Others (specify)         

28.  Have you ever had to pay for IUD service?

                       i.          Yes

                      ii.          No      

29.  If yes, how much?

                       i.          <100 INR

                      ii.          100-500 INR

                     iii.          >500 INR

30.  How far is the nearest healthcare facility from your residence?

                      i.          0-3 km

                     ii.          4-6 km

                    iii.          7-10 km

                    iv.          >10 km

31.  Would you prefer to use an IUD in the future

                     i.          Yes

                    ii.          No

                   iii.          Not sure

II. Qualitative questionnaires

In-Depth Interview Questionnaires for Eligible Women/Mothers

Ice breaker

Section I: Information

1.     How many children do you have?

2.     Are you planning to have more children?

3.     How long are you planning to wait before having another child?

4.     How old is your youngest child?

5.     Is there any health awareness regarding birth spacing in your community?

6.     If yes, what do you know about it?

7.     According to you, what is the reason for using contraceptive methods? (*For birth spacing or limiting births*)

8.     Why is birth spacing important?

9.     What types of contraceptive methods do you know? (*If IUD is not mentioned, go to Question 10; if mentioned, skip Question 10.*)

10.  Do you know about the IUD? (*Please Elaborate*)

11.  If yes, from where did you know about it?

12.  Do you know who is eligible to use an IUD?

Section II: Service Delivery

1.     Have you used any contraceptive method?

2.     If yes, which type of contraceptive method do you prefer to use?

3.     Who helped you in accessing the contraceptive service?

4.     If you are using Condom/OCP/Injectables/IUD, why did you choose this method?

5.     If you are not using any contraceptive method, what is the reason? Would you like to use one in the future?

6.     If you are using an IUD, how did you come to this decision?

7.     What is your spouse’s opinion regarding contraceptive use?

8.     Which type of IUD are you using?

9.     At which facility was the IUD inserted?

10.  For how long have you been using it?

11.  If you stopped using it, what were the reasons for stopping?

12.  If you experienced any medical problems, what was your immediate action?

13.  Before IUD insertion, did you receive counseling?

14.  Have you faced any challenges in accessing contraceptive services?

Section III: Health Workforce

1.     Do health workers visit your home to provide information about the IUD?

2.     If No, did you visit any health facility to enquire about contraception?

Section IV: Medical Product

1.     Did you experience any problems while using the IUD? If yes, please elaborate.

2.     Have you faced any difficulties in availing IUD services from health centers?

3.     Did you have to visit multiple health facilities to obtain the service?

Section V: Finance

1.     Did you ever have to pay for IUD insertion or related services?

2.    If yes, how much did you spend?

Section VI: Leadership

1.     Have you ever recommended the IUD to anyone?

2.    According to you, what suggestions would help improve IUD uptake? What steps should the health system take to provide better services?

In-Depth Interview Questionnaires for ASHAs

Ice Breaker

Section I: Information

1.     Have you received training on family planning (FP) counselling? If yes, how often? Do you attend all the training sessions?

2.     Does the health department conduct awareness programmes on the IUD? If yes, how often?

3.     According to you, what do you know/understand about Family planning and birth spacing?

4.     Which contraceptive methods are you allowed to prescribe? How do you procure these products? What challenges did you face in procuring them?

5.     Which family planning methods are used or preferred by women in your locality?

         a. Why Condoms/OCPs/Injectables/IUD?

         b. Why not OCPs/Injectables/IUD? (*If the IUD was not mentioned*)

6.     Do you know about the Intrauterine Contraceptive Device (IUD)?

7.      If yes, can you explain more about it and its purpose?

8.     How often do you provide information or conduct awareness activities on family planning/IUD?

9.     What is people’s perception of the IUD?

10.    Do women report any complaints or face challenges with any contraceptive methods?

Section II: Workforce

1.     Have you received any training on IUD counselling?

2.     Who provides IUD counselling at your centre?

3.     Are you always present during the counselling sessions?

4.     Do health workers provide proper explanations about this contraceptive method?

Section III: Service Delivery

1.     Who performs IUD insertion?

2.     How many women in your area use the IUD?

3.     Do you maintain records of IUD users or insertions?

4.     How do you report IUD-related data?

Section IV: Medical Product

1.     What other family planning methods are used or preferred by women in your locality, and why?

2.     Do you have any information regarding the quality of the IUD or other contraceptive products?

Section V: Finance

1.     Do you know about the financial repercussions of using an IUD?

2.     Is there any cost associated with IUD services for patients?

Section VI: Leadership

1.     Have you faced any difficulties in contacting appropriate authorities regarding day-to-day challenges?

2.      In the past, if there were any glitches in the system, have you had any problem in conveying these to the appropriate authorities?

3.     To whom have you referred to opt for an IUD?

4.     What suggestions will you give on how to increase IUD uptake in the community?

In-Depth Interview Questionnaires for Nursing Supervisors/Staff Nurses

Ice Breaker

Section I: Information

1.   What is the estimated population served by this facility?

2.   In your estimation, how frequently do people from outside this facility area seek care here? 

3.   What is the antenatal care coverage rate for this facility in the previous year?

4.   What was the delivery coverage rate for this facility in the previous year?

5.   What was the postpartum care coverage rate for this facility in the previous year?

6.   What types of client information, education and communication (IEC) materials are available for FP services?

7.   How are unmet needs for family planning among postpartum women during the first year postpartum?

      a.   Higher than the unmet need for other women

      b.   Same as the unmet need of other women

      c.   Lower than the unmet need for other women

8.   What are the reasons for expulsion of the postpartum IUD?

      a.   Choosing the correct type of IUD will reduce expulsion rates.

      b.   Proper insertion technique reduces the expulsion rates.

      c.   Expulsion rates are lower in low-party women.

9   What is NOT an acceptable time to insert an IUD immediately postpartum?

      a.   20 minutes after expulsion of placenta  

      b.   36 hours postpartum

      c.   2 weeks postpartum

Section II: Service Delivery

1.   Have you received training on FP counselling?

2.   Do you regularly counsel pregnant or postpartum women on FP?

3.   When do you counsel pregnant or postpartum women on FP? 

4.   Have you received training on IUD insertion?

5.   Have you ever inserted an IUD? If yes, how many IUDs did you insert in the past one month?

6.   Of those, how many were post-placental (within 10 min after delivery), intra-caesarean, immediate post-delivery (within 48 hours of delivery), or extended postpartum (4 Weeks post-delivery)

7.   If not inserted, what is your main reason?

8.   In your experience, when is the best time to counsel pregnant or postpartum women on PPIUD?

9.   When do pregnant or postpartum women make their decision about FP in general?

10. What are the complication case reports by the client following IUD insertion?

11.   What step to be taken to mitigate the problem?

Section III: Medical Product

1.   How are the quantities of IUD and medical supplies needed by this facility determined? 

     a.   Quantities determined at the national level (*e.g., standard drug kit*)

     b.   Quantities determined at the district level

     c.   Quantities determined through supervisory visits

     d.   Quantities determined by facility staff

     e.   Other (specify): _________

2.   Have you ever faced any challenges in availing of equipment/products? 

3.   How long does the procuring process take?

4.   Is there a time you faced a shortage of supply?

5. Are FP clients required to purchase/provide supplies like gloves, IUDs, and antiseptic solutions?

Section IV: Leadership

1.   What steps are taken by your facility to improve contraceptive services?

In-Depth Interview Questionnaires for Medical Officers

Ice Breaker

Section I: Information

1.   What is the estimated population served by this facility?

2.   In your estimation, how frequently do people from outside this facility area seek care here?

3.   What is the antenatal care coverage rate for this facility in the previous year?

4.   What was the delivery coverage rate for this facility in the previous year?

5.   What was the postpartum care coverage rate for this facility in the previous year?

6.   What types of client information, education and communication (IEC) materials are available for FP service?

7.   How are client’s opinion on IUD? 

8.   What are the major reasons for low uptake?

9.   How can the health system improve the FP services?

Section II: Service Delivery

1.   How many staff at this facility have received training in Family Planning (*IUD insertion Skills, Family Planning counselling/continuing education*) within the past twelve months?

2.   How many times are family planning counselling and services provided in a month at this facility?

3.   Is a skilled attendant for maternity services available on site, 24 hours/day, 7 days/week? By “Skilled attendant” like a physician, Health officer, midwife, or nurse.

4.   What are the types of FP service delivery guidelines/clinical management protocols given for the service provider?

5.   Are these guidelines/protocols available for reference by staff at all times? If NO: Why not?

6. How many IUCD insertions did you receive in a month? If none, what may be the reason?

7.   Of those, how many were post-placental (within 10 min after delivery), intra-caesarean, immediate post-delivery (within 48 hours of delivery), or extended postpartum (4 Weeks post-delivery)?

8. What are the complication case reports by the client following IUD insertion?

9.   What step to be taken to mitigate the problem?

Section III: Medical Product

1.   How are the quantities of IUD and medical supplies needed by this facility determined? 

      a.   Quantities determined at the national level (*e.g., standard drug kit*)

      b.   Quantities determined at the district level

      c.   Quantities determined through supervisory visits

      d.   Quantities determined by facility staff

      e.   Other (specify): _________

2. How long does the procurement process take?

3   Have you ever faced any challenges in availing equipment or products?

4.   Have you faced a shortage of supply in the facility?

5. Are FP clients required to purchase or provide supplies like gloves, IUDs, and antiseptic solutions?

Section IV: Leadership

1.   What steps are taken by your facility to improve contraceptive services?

Questionnaires for Focus Group Discussion

Ice Breaker

1.   Why do you think birth control is important?

2.   Who first taught you about birth control? What do you call it?

3.   What birth control methods do you know of?

4.   How comfortable are you talking about birth control? With friends and family? With your healthcare provider?

5.   Did the health workers give any awareness on family planning (FP)?

6.   What is your spouse’s opinion on family planning (FP)?

7.   When do women prefer to have children and why?

8.   How does the group feel about using modern contraceptive methods compared to traditional methods?

9.   Do you think family planning helps financially in your household? If yes, how?

10.   Why did each of you choose your particular contraceptive methods? If you shifted from an IUD to another method, what was the reason?

11.   Do you think contraceptives can make you barren? 

12.   For those who have used an IUD, what were your experiences and opinions?

13.   Before receiving family planning services, did the service providers ask about your medical history or perform any tests?

14.   Before IUD insertion, did you receive proper counselling? Were you given detailed information about the IUD (*including duration of use, mechanism of action, benefits, possible side effects, insertion, removal, and other considerations*)?

15.   Did any of you receive any incentives for using the contraceptive method you chose? (*Monetary or non-monetary*).

16.   Did any of you notice any changes in your menstrual cycle while using any particular contraceptives?

17.   After IUD removal, how was your fertility affected, if applicable?

18.   How frequently do you visit a healthcare facility for IUD-related check-ups?

19.   Do you feel like your lifestyle/daily routine was affected by using an IUD?

20.   Do you face any challenges in obtaining any of the contraceptives when needed?

21.   What steps do you think the health care providers should take to improve IUD uptake in the community? Please elaborate.
